# Obstructive sleep apnoea, sleep bruxism and gastroesophageal reflux – mutually interacting conditions? A literature review

**DOI:** 10.1111/adj.13042

**Published:** 2024-10-21

**Authors:** Davis C. Thomas, Anna Colonna, Daniele Manfredini

**Affiliations:** ^1^ Rutgers School of Dental Medicine Newark New Jersey USA; ^2^ School of Dentistry, Department of Medical Biotechnologies University of Siena Siena Italy

**Keywords:** Obstructive sleep apnoea, sleep bruxism, masseter muscular activity, gastroesophageal reflux disease, orofacial pain, temporomandibular disorders

## Abstract

The purpose of the present manuscript is to provide an overview for researchers and clinicians summarizing the knowledge concerning the relationship between some of the main sleep‐related conditions of dental interest: sleep bruxism (SB), obstructive sleep apnoea (OSA) and gastroesophageal reflux disease (GERD). Starting with the discussion of the evolving current knowledge on SB, the interconnections are discussed. Most of the available literature focused on the possible relationship between OSA and SB, but a clear pathophysiological connection or temporal relationship has not been identified. Despite the paucity of data on the possible commonalities, SB, OSA and GERD constitute a complex network of conditions that may affect the clinical and research dental practice, and they are rarely found in isolation. In this scenario, the key role of dental practitioners as sentinel in the case of these sleep‐related conditions is important, thanks to their ability and the knowledge to identify signs, symptoms and risk factors that are signs of ongoing sleep‐related conditions. Thus, dental practitioners are recommended to view bruxism as a potential gateway to medicine.

**Abbreviations and acronyms**
AB: awake bruxism
EMG: electromyographic
GERD: gastroesophageal reflux disease
MMA: masticatory muscle activity
OFP: orofacial pain
OSA: obstructive sleep apnoea
PSG: polysomnography
SB: sleep bruxism
TMD: temporomandibular disorders

**Clinical Relevance**

From a research and clinical viewpoint, sleep bruxism, obstructive sleep apnoea and gastroesophageal reflux disease establish a multifaceted network of conditions that are rarely found in isolation and may affect the dental practice. It is crucial for clinicians and researchers to deepen their knowledge on these sleep‐related conditions. The aim of the present manuscript is to present a narrative overview to help dental practitioners understand their role as potential sentinels of the stomatognathic system when sleep‐related conditions of potential systemic impact are present.

## INTRODUCTION

Sleep bruxism (SB), obstructive sleep apnoea (OSA) and gastroesophageal reflux disease (GERD) are sleep‐related phenomena that are gaining increasing attention in several disciplines, such as dentistry, psychology, neurology, gastroenterology and sleep medicine.^1^ They are of interest for dental professionals as they can affect the prognosis of dental treatment and may also have an influence on orofacial pain, so that dental practitioners can play a key role in their diagnosis and/or management.^2^ As a recognition of this interconnection, it is remarkable that the American Academy of Orofacial Pain introduced sleep in its mandate^3^ and the Australasian Academy of Dental Sleep Medicine presented orofacial pain (OFP) and temporomandibular disorders (TMD) as dental sleep‐related conditions.^4^

Given the aetiological complexity and the variegate clinical picture of these phenomena, as well as the possibility that they occur simultaneously in the same patient, their management requires a multidisciplinary approach, with a tight cooperation between dentists and different medical specialists. The former have a potential sentinel role in the screening and early recognition of individuals with sleep‐related conditions and must manage the possible consequences in the oral cavity and the other stomatognathic structures; the latter have specific competences for the diagnosis and treatment of OSA and GERD.^2^

Within this framework, the present manuscript summarizes current knowledge regarding sleep bruxism, with focus on the ongoing paradigm change viewing bruxism as ‘a sign of some underlying or associated conditions,’ which is used to explore the possible correlation with obstructive sleep apnoea and gastroesophageal reflux disease.

## SLEEP BRUXISM

Sleep bruxism is a masticatory muscle activity (MMA) during sleep that is characterized as rhythmic (phasic) or non‐rhythmic (tonic) and is not a movement disorder or a sleep disorder in otherwise healthy individuals.^5^ In accordance with this definition, which was provided in 2018 by an expert consensus panel and should be viewed as the standard of reference to standardize language,^6^ SB must be distinguished from awake bruxism (AB), which may have different aetiology, comorbidities and clinical consequences because of the different spectrum of muscle activities that may feature the various activities in relation to the circadian manifestation.^6^, ^7^, ^8^, ^9^, ^10^

SB is not a disorder per se in otherwise healthy individuals, but it might be viewed as a potentially non‐harmful condition that can be a risk and/or sometimes even potentially protective factor for different clinical consequences, independently on any specific neurological correlations.^6^, ^7^, ^11^, ^12^, ^13^, ^14^, ^15^, ^16^ As pointed out in a couple of short commentaries and explanatory notes, confusion arose in the literature based on the assumption that bruxism can be ‘diagnosed’ only with polysomnography (PSG).^12^, ^14^ Whilst this is surely the best possible way to investigate the masseter activity within the context of sleep architecture, it has been repeatedly noted that SB/PSG diagnostic criteria are just based on the count of arousal‐related rhythmic masseter muscle activity (RMMA) events. This offers a partial view of the bruxism picture, in relation with the fact that other activities included in the definition, such as prolonged teeth clenching and mandible bracing in particular, are excluded from the assessment. This premise is fundamental to understand that most information on the relationship between SB and other conditions is influenced by the diagnostic approach that is adopted in a certain specific investigation. For instance, Manfredini and Lobbezoo have often discussed how the literature on bruxism and temporomandibular disorders (TMDs) may or may not find a relationship between the two conditions depending on the approach to bruxism evaluation, with inconsistent results obtained in PSG studies and vice versa a solid association described in self‐reported studies, possibly because of the inclusion of clenching and bracing report.

Among the potential protective effects of SB, a reduced risk of detrimental chemical tooth wear by increasing salivation in case of gastroesophageal reflux^17^ and/or an increased upper airway patency that would aid in the prevention of the airway collapse leading to obstructive sleep apnoea have been hypothesized.^18^, ^19^ On the other hand, the potential negative clinical consequences in the dental field include repeated fractures of teeth, dental restorations and implants,^20^, ^21^ intrinsic mechanical tooth wear (attrition),^22^ and, as anticipated above, temporomandibular disorders.^23^, ^24^ Based on the above consideration, a recent paper by the core authors of the consensus papers on bruxism definition suggested that bruxism cannot be viewed as *the* disorder in any case. Indeed, even in cases of severe bruxism and the presence of clinical consequences, bruxism itself should be viewed as a sign of an associated or underlying condition, rather than the actual disorder itself.^12^ Such construct is important to transfer to general dentists, whose knowledge is still permeated with information on past theories.^25^, ^26^

The evolution in bruxism knowledge led the international expert panel to propose a Standardized Tool for the Assessment of Bruxism (STAB), which is based on the collection of information from multiple sources (i.e. subject, clinician, instruments) to gather data on bruxism aetiology, status, consequences and comorbid conditions.^27^, ^28^ The instrumental measurement of electromyographic (EMG) activity in the natural environment during sleep seems to be the most appropriate approach available to collect information on the different SB behaviours^29^, ^30^; however, to date, these type of devices, in order to be used routinely in a clinical setting, must be improved both in terms of software and hardware.^31^

## EPIDEMIOLOGY OF SB AND OSA

Based on sleep architecture investigations, the prevalence of SB is high in children,^32^ reduces to about 8%–16% in adults and has an additional decrease with age.^7^, ^33^ As a remark, it must be noted that most available information is drawn either from a few PSG studies based on the above‐cited arousal‐related SB criteria or large‐scale unspecific self‐reported investigations. Thus, the actual frequency of the full spectrum of bruxism activities is still to be elucidated. In contrast, significant research has been carried out into the epidemiology and effects of OSA due its serious health effects.^34^, ^35^

It is thus intuitive that OSA, a chronic sleep‐related breathing disorder, may represent a serious public health problem due to its considerable prevalence at the general population level.^34^, ^35^ In addition, a huge number of patients remain undiagnosed or receive a diagnosis only after the manifestation of the long‐term consequences (e.g. cardiovascular diseases, diabetes mellitus and neurocognitive disorders).^36^, ^37^, ^38^

From an epidemiological point of view, it is interesting to note that patients with OSA have a significantly higher SB prevalence than the general population,^39^, ^40^, ^41^, ^42^ with reported values ranging from 26% to 53.7%.^39^ A large‐scale investigation on sleep bruxism based on a full polysomnographic recording was performed by Li *et al*., who found that the prevalence of SB in patients with OSA was 49.7%,^40^ whilst Tan *et al*. reported that one‐third (33.3%) of patients with OSA had PSG‐diagnosed SB.^41^

This highlights that aside from the higher prevalence of SB among patients with OSA compared with the general population, subjects with severe apnoea tend to have higher bruxism indices when compared to patients with mild and moderate apnoea.^19^ At the same time, the apnoea and hypopnoea events (AHE), characterized by total (apnoea) or partial (hypopnoea) airway obstruction, are related with arousals. In turn, SB has often been associated with arousals^5^, ^11^, ^41^, ^43^ in response to respiratory efforts.^44^

## RELATIONSHIP BETWEEN SB AND OSA

Based on the above observations, the possible temporal and causal association between OSA and SB has received much attention in recent years. In 2015, Manfredini *et al*. hypothesized the existence of different possible scenarios for a temporal relationship between the two conditions: (1) the two phenomena are unrelated; (2) the onset of the OSA event precedes the onset of the SB event by a few seconds; (3) the onset of the SB event precedes the onset of the OSA event by a few seconds, and (4) the onset of the OSA and SB event occurs at the same moment.^18^ The authors suggested that in some cases, SB may have a protective role for OSA patients, based on the hypothesis that the activation of masseter muscles may contribute to pull the tongue forward during mouth closure, causing an advancement of the jaw, an increase of upper airway patency and an interruption of respiratory event.^18^

Whilst some case reports and small case series on the topic had already been published, that paper sparked the attention on the need to get deeper into the assessment of the possible correlation between OSA and SB. Other papers investigating the possible correlation between OSA and SB reported inconsistent results.^45^, ^46^, ^47^, ^48^ For instance, Saito *et al*. reported that respiratory events (i.e. apnoea and hypopnoea events) do not correlate temporally with sleep bruxism events (SBEs), in disagreement with their own previous results.^46^ These data are in line with a previous investigation conducted by Sjoholm et al. that reported that only 3.5% of SBEs are directly associated with the AHE in a group of patients with moderate OSA and 14.4% in a mild OSA group.^47^ On the contrary, Philipps *et al*. suggested a positive association between OSA and SB,^45^ in accordance with an earlier study by Saito *et al*., that investigated the temporal relationship between AHE and SB‐related rhythmic masticatory muscular activity (RMMA) using a 5‐min time window.^48^

A more recent investigation proposes that, whilst from a global point of view there is no correlation between OSA and SB, if the different types of respiratory events are considered separately (i.e. obstructive apnoea, central apnoea, mixed apnoea and hypopnoea), SBEs occurred with different temporal sequences. The majority of SBEs (66%) were noted to occur after obstructive apnoea (OA), whereas 84% of SBEs occurred prior to central apnoea (CA).^19^ These data are in line with Tsujisaka *et al*., who discovered that most of the CA events occurred after the SBE.^49^

In addition, some authors suggested that microarousals that occur during AHEs maybe the main cause of SB.^50^ The proposed pathophysiology is that intermittent hypoxia resulting from OSA causes an arousal and then the RMMA, that is typical of SB, is produced to minimize the drop in blood oxygen levels.^51^ However, the same authors suggested a positive correlation between sleep Bruxism Episode Index (BEI) and Apnea Hypopnea Index (AHI) only in the group with mild and moderate OSA, but not in severe OSA (AHI ≥30), thus specifying that the OSA severity influences the correlation between BEI and AHI.^46^ In contrast, Colonna *et al*. found that SB tends to increase in relation with the increase in AHI severity, even if the data are incomparable due to the different study design.^19^ Finally, Li *et al*.^40^ report that nearly half of adults with OSA have comorbid SB.

In view of this, several authors support the complexity of the OSA‐SB relationship and the probably limited role of SB as a purely protective factor, especially in severe OSA. An explanation could be that SBE events are part of the cascade of events and neurovegetative changes that occur during respiratory arousals, rather than hypothesizing a direct relationship with RE.^51^, ^52^

Thus, the results of different investigations appear to be inconsistent, highlighting the need for further research to characterize the relationship between SB and respiratory events during sleep.^51^, ^53^, ^54^ This could be partly explained also with the fact that the relative predominance of a specific sequence of events may vary at the individual level. Comparing findings between studies was further impacted by the lack of standardized data collection strategies, particularly with regard to the time frame in which events would be considered to be associated with each other.^19^

For these reasons, future research should focus on the existence of possible patients' phenotypes in which SB may represent a protective factor against OSA or be part of an OSA‐related arousal. This could ideally lead to end typing bruxism as far as the underlying physiopathology is concerned. In particular, an evaluation of the anatomical features of OSA patients could be carried out, with the aim of identifying subjects who present a potentially protective SB due to the fact that a mandible advancement reflex interrupts the apnoea. It might be hypothesized that in OSA patients with an obstruction of the upper airways, viz., at the oropharyngeal level, the AHE could induce respiratory efforts producing a cascade of events that ends with an increase in the muscle activity and may result in mandibular protrusion and subsequent opening of the upper airways.^18^, ^19^ Future studies should further explore this aspect, especially considering that if this correlation was confirmed, SB would be seen as a potential clinical predictor of OSA in some individuals. Contextually, the dentist could act as a medical sentinel, and the treatment of OSA could be seen as a causal approach to manage the clinical consequences of SB (e.g. tooth wear, orofacial pain).^55^ This hypothesis is supported by the concept that the use of MAD simultaneously decreases AHI and SBI and that MADs are differently effective with respect to the facial morphology of patients.^56^, ^57^, ^58^, ^59^

All the above aspects must be considered in the light of the need to concurrently adopt the current definition of bruxism and to move on from the adoption of cut‐off points to discriminate between bruxers and non‐bruxers, embracing an evaluation based on the continuum of jaw muscles activities.^5^, ^7^, ^11^, ^18^ On this purpose, a fascinating hypothesis might be that OSA individuals who do not have any event during periods of prolonged activation of the masseter are actually keeping the mandible protruded with consequent maintenance of airway patency.

## GASTROESOPHAGEAL REFLUX DISEASE

In this scenario, other habits, conditions and factors that are emerging as correlated with both SB and OSA must also be taken into account, such as gastroesophageal reflux.

Gastroesophageal reflux disease (GERD) is a chronic gastrointestinal condition characterized by the regurgitation of gastric contents into the oesophagus, which in high‐income countries affects approximately 20% of the adult population.^60^ The pathophysiology of GERD is multifactorial, involving the influence of the tone of the lower oesophageal sphincter, oesophageal motility and oesophageal mucosal defence against the refluxate and the presence of a hiatal hernia.^61^ The various processes lead to the disruption of the oesophagogastric junction barrier, resulting in exposure of the oesophagus to acidic gastric contents.^62^

This disorder is of great interest in the dental field as well, as it can cause severe dental erosion resulting from intrinsic chemical tooth wear accompanying the typical battery of medical symptoms (e.g. heartburn, regurgitation, chest pain, chronic cough, laryngitis or asthma).^63^ As in the case of OSA, some authors hypothesized that tooth wear and GERD symptoms severity are related.^22^, ^64^ Some papers suggest the existence of a possible association between SB and GERD,^17^, ^65^, ^66^ which would lead to a more rapid loss of tooth substance due to the fact that grinding and/or clenching on teeth covered by acid may accelerate the speed of hard tissue loss.^66^ Increased temporomandibular disorder prevalence also appears to be associated with GERD.^67^, ^68^ Nonetheless, the literature is unfortunately still scarce to draw any conclusions concerning the actual SB/GERD relationship.

The interconnection between the three conditions (i.e. SB, OSA and GERD) seems to be suggested by the fact that GERD would relate to a worsening in OSA severity.^69^ Ex juvantibus information gathered from treatment studies might be useful in the future to understand whether the conditions share common mechanisms related with sleep arousals and may respond to the same approaches, such as behavioural approaches and strategies stabilizing sleep quality. Within this framework, it might be suggested that, being OSA and GERD are medical conditions that, contrarily to the potentially harmless SB, require immediate medical treatment due to the possible adverse consequences on the general health, treatment studies may indeed provide useful information to understand the commonalities.

## CONCLUSIONS

As a summary, based on the available data, there seem to be more speculations than facts as far as the interconnection between SB, OSA and GERD is concerned. There is not enough scientific evidence yet to define a clear pattern of epidemiological and temporal relationship, if any, between SB, OSA and GERD. Similarly, the potential protective role of SB in patients with OSA and/or GERD remains uncertain. However, from a clinical and research viewpoint, SB, OSA and GERD constitute a complex network of conditions that may affect the dental practice and are rarely found in isolation.^70^

In this scenario, the key role of dental practitioners as sentinel in the case of these sleep‐related conditions (i.e. SB, OSA and GERD) is crucial, thanks to their ability and the knowledge to identify signs, symptoms and risk factors that are signs of ongoing sleep‐related conditions. Thus, dental practitioners are recommended to view bruxism as a potential gateway to medicine.^71^

## CONFLICT OF INTEREST

All authors certify that they have no affiliations with or involvement in any organization or entity with any financial interest (such as honoraria; educational grants; participation in speakers' bureaus; membership, employment, consultancies, stock ownership or other equity interest; and expert testimony or patent‐licensing arrangements) or non‐financial interest (such as personal or professional relationships, affiliations, knowledge or beliefs) in the subject matter or materials discussed in this manuscript.

## FUNDING INFORMATION

No funding was received for this research.

## AUTHOR CONTRIBUTIONS

**DC Thomas:** Writing – original draft; writing – review and editing. **A Colonna:** Writing – original draft. **D Manfredini:** Supervision; writing – review and editing.
